# SHG-specificity of cellular Rootletin filaments enables naïve imaging with universal conservation

**DOI:** 10.1038/srep39967

**Published:** 2017-01-06

**Authors:** Toshihiro Akiyama, Akihito Inoko, Yuichi Kaji, Shigenobu Yonemura, Kisa Kakiguchi, Hiroki Segawa, Kei Ishitsuka, Masaki Yoshida, Osamu Numata, Philippe Leproux, Vincent Couderc, Tetsuro Oshika, Hideaki Kano

**Affiliations:** 1 Department of Applied Physics, Graduate School of Pure and Applied Sciences, University of Tsukuba, 1-1-1 Tennodai, Tsukuba, Ibaraki 305-8573, Japan; 2 Division of Biochemistry, Aichi Cancer Center Research Institute, 1-1 Kanokoden, Chikusa-ku, Nagoya, Aichi 464-8681, Japan; 3 Department of Ophthalmology, Faculty of Medicine, University of Tsukuba, 1-1-1 Tennodai, Tsukuba, Ibaraki 305-8575, Japan; 4 Ultrastructural Research Team, RIKEN Center for Life Science Technologies, 2-2-3 Minatojima-minamimachi, Chuo-ku, Kobe, Hyogo 650-0047, Japan; 5 Department of Cell Biology, Tokushima University Graduate School of Medical Science, 3-18-15 Kuramoto-cho, Tokushima 770-8503, Japan; 6 Department of Chemistry, School of Science, The University of Tokyo, 7-3-1 Hongo, Bunkyo, Tokyo 113-0033, Japan; 7 Faculty of Life & Environmental Sciences, University of Tsukuba, 1-1-1 Tennodai, Tsukuba, Ibaraki, 305-8572 Japan; 8 Institut de Recherche XLIM, UMR CNRS No. 7252, 123 avenue Albert Thomas, 87060 Limoges CEDEX, France; 9 Institute of Applied Physics, University of Tsukuba, 1-1-1 Tennodai, Tsukuba, Ibaraki 305-8573, Japan; 10 Tsukuba Research Center for Interdisciplinary Materials Science (TIMS), University of Tsukuba, 1-1-1 Tennodai, Tsukuba, Ibaraki 305-8571, Japan

## Abstract

Despite growing demand for truly naïve imaging, label-free observation of cilium-related structure remains challenging, and validation of the pertinent molecules is correspondingly difficult. In this study, in retinas and cultured cells, we distinctively visualized Rootletin filaments in rootlets in the second harmonic generation (SHG) channel, integrated in custom coherent nonlinear optical microscopy (CNOM) with a simple, compact, and ultra-broadband supercontinuum light source. This SHG signal was primarily detected on rootlets of connecting cilia in the retinal photoreceptor and was validated by colocalization with anti-Rootletin staining. Transfection of cells with Rootletin fragments revealed that the SHG signal can be ascribed to filaments assembled from the R234 domain, but not to cross-striations assembled from the R123 domain. Consistent with this, Rootletin-depleted cells lacked SHG signal expected as centrosome linker. As a proof of concept, we confirmed that similar fibrous SHG was observed even in unicellular ciliates. These findings have potential for broad applications in clinical diagnosis and biophysical experiments with various organisms.

Modern microscopy was developed with a great deal of assistance from molecular spectroscopy, which provides distinct image contrast resulting from the interactions of molecules with light^1^. Fluorescence microscopy is among the most popular imaging methods for biological studies. In particular, in immunofluorescence microscopy, the combination of target-specific antibodies and fluorescent probes confers exquisite specificity and unprecedented sensitivity. However, many fluorescent labels perturb bioactive molecules such as signaling peptides, metabolites, and structural proteins, and these perturbations can distort experimental results. Moreover, label-free medical diagnostics is favorable to achieve the minimally invasive procedure. Thus, label-free molecular contrast with spectroscopic specificity is ultimately indispensable for biological and medical imaging.

A novel approach for label-free imaging, coherent nonlinear optical microscopy (CNOM), has recently emerged as a powerful molecular imaging tool that can detect non-fluorescent species in live cells or tissues. Coherent interactions of molecules with radiation give rise to in-phase signal radiation from the irradiated molecules. The total signal radiation is thus the vector sum of these in-phase signal radiations, leading to amplification of the signal by several orders of magnitude^2^,^3^. A well-established CNOM technique is second harmonic generation (SHG) microscopy^4^,^5^,^6^,^7^,^8^. SHG is a frequency-doubling nonlinear optical process, which originates from coherent interaction of laser pulses with organized ensembles of non-centrosymmetric targets with significant hyperpolarizability^4^,^9^. Also widely used are coherent Raman scattering (CRS) microscopy, such as coherent anti-Stokes Raman scattering (CARS)^10^,^11^, and stimulated Raman scattering (SRS)^12^,^13^ microscopy, which provide molecular contrast through the characteristic vibrational frequencies of specific chemical bonds. In contrast to a fluorescence process, coherent interactions do not require direct photoexcitation; consequently, CNOM does not require any fluorophores and can provide label-free molecular images.

In addition to label-free imaging capability, CNOM methods have unique inherent advantages for naïve imaging. (1) High sensitivity. Due to the coherent summation of signal radiation, CNOM boosts signal intensity to achieve highly sensitive imaging and provide natural endogenous image contrast. Moreover, because CNOM does not require photoexcitation of molecules, it conserves incoming energy and preserves the coherence of incident laser radiation. (2) Non-toxicity. CNOM does not suffer from phototoxic effects or photo-bleaching, both of which are often critical for one- and multi-photon excitation fluorescence microscopy. (3) Spatial resolution. Due to its nonlinear optical properties, the signal is restricted to the focal volume (~1 μm^3^), resulting in inherent three-dimensional sectioning capability. (4) Background free. Since the wavelength of the signal is different from (typically shorter than) those of the incident laser, there is no unwanted background (Fig. S1). (5) Multimodality. Owing to recent advancements in supercontinuum (SC) generation technology^14^, ultra-broadband excitation has been achieved in CNOM, which enables us to launch unique and multi-color nonlinear optical processes such as multiplex sum frequency generation (SFG)^15^, third-order sum frequency generation (TSFG)^16^, and CARS as well as monochromatic SHG and third harmonic generation (THG) (See also Fig. S1). These methods are complementary to one another, and their utility is further increased by integrating them into a multimodal microscopic unit.

Among CNOMs, SHG microscopy provides unique image contrast due to its even (second)-order nonlinear optical susceptibility. As described earlier, the key harmonophores for SHG signals are highly polarizable and have well-ordered non-centrosymmetric molecular organizations or structures with SHG-active molecules^5^. To date, collagens^17^, mitotic spindles^5^, contractile filaments in muscle^5^, muscle myosin^18^, and axons^19^ have been used as endogenous harmonophores to visualize specific molecular structures. However, the SHG-active structures reported to date generally constitute large architectures either insider or outside of cells. Smaller cytoskeletal organelles have not yet been studied in this manner.

The cilium, a tiny protrusion from cell surface, is a biologically important organelle that functions as a cellular ‘antenna’^20^. This structure is commonly based on a microtubule-based axoneme originating from the distal tips of basal body, analogous to the mother centriole^21^. The clinical importance of cilia *in vivo* has been recognized since the mid-1990s. Hereditary dysfunctions of cilia cause a spectrum of morphological and mental disorders, named ciliopathies, with complex symptoms including retinopathy, situs inversus, polydactyly, kidney disease, mental retardation, and obesity^22^,^23^,^24^,^25^,^26^. These disorders are primarily the consequence of failure to sense diverse extracellular signals and transduce them into cellular responses via cilia, supporting the idea that cilia play important roles in signal transduction.

A cilium in a cultured diploid cell is observed as an immotile single protrusion, termed a “primary cilium,” first described in the 19th century^27^. The primary cilium has distinct kinetics that are the inverse of the cell division cycle: it is assembled only during cellular quiescence, but does not grow during cell cycle progression^28^,^29^,^30^. The molecular mechanisms underlying these kinetics have been partially elucidated^31^,^32^.

The ciliary rootlet, on which we focus in this study, is an anatomical structure first discovered in 1880^33^; it consists of a fibrous cytoskeletal organelle that links the base of the cilium to the cell body, indicating a role in structural assurance and signal mediation. Across species, rootlet ultrastructure consists of filaments with distinct cross-striations at intervals of 50–70 nm^34^. Recently, a molecular cloning and genetic approach identified Rootletin, a novel coiled-coil protein, as a structural component of the ciliary rootlet in murine retinal photoreceptor cells^35^. Genetically Rootletin-depleted mice, which lack rootlets, exhibit photoreceptor degeneration and impaired mucociliary clearance in multi-ciliated trachea over time, consistent with the function of Rootletin in providing long-term structural support for the cilium^36^.

Rootletin is functionally conserved across species. The *Caenorhabditis elegans* Rootletin orthologue, CHE-10, also supports cilia^37^. In *Drosophila melanogaster*, Root is the sole orthologue of mammalian Rootletin and C-Nap1. Root mutants also lack ciliary rootlets and exhibit impaired neuronal response to mechano- and chemosensation, including negative geotaxis, taste, touch, and sound^38^,^39^. Thus, Rootletin is thought to be engaged in general sensory perception, structurally supporting the cilium or mediating mechano- and chemosensation.

Independently, Rootletin was also detected in cultured cells^40^, in which it forms centriole-associated fibrous structures^24^. In particular, depletion of Rootletin by small interfering RNA (siRNA) results in centrosome splitting, indicating that Rootletin filaments contribute to establishment of centrosome cohesion before mitosis. Overall, Rootletin filaments contribute not only to supporting sensory cilia but also to centrosome integrity in cultured cells, increasing their value as research targets and the demand for naïve imaging.

We successfully performed selective imaging of ciliary rootlet-composing Rootletin filaments, in retina in frozen sections of a rat eye, in cultured cells, and even in unicellular ciliates, using the SHG channel equipped on our multimodal CNOM. Transfection of exogenous Rootletin fragments revealed that the SHG signal could be ascribed to filaments assembled from the Rootletin R234 domain. Consistent with this, Rootletin-depleted cultured cells lacked the SHG signal on centrosomes, where endogenous cellular Rootletin forms filaments involved in centrosome cohesion. Thus, we validated the SHG signal from endogenous Rootletin filaments, which has broad potential for use in clinical diagnosis and biophysical experiments even in distant species.

## Results

### Truly label-free CNOM visualizes hierarchical structures of retinal layers, highlighting novel filamentous layers defined by SHG

**Figure 1:**
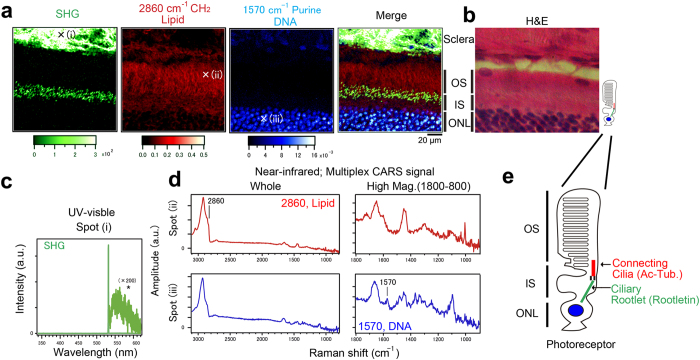
Truly label-free penta-modal CNOM visualizes hierarchical structures of retinal layers, highlighting novel SHG-indicated filamentous layers. Spectroscopic imaging was performed with fixed rat retina using our custom microscope. **(a)** A collection of label-free images of SHG (green) and CARS at 2860 cm^−1^ (red) and 1570 cm^−1^ (blue), showing the distributions of harmonophores, lipid, and DNA, respectively. Note the unexpected filamentous layer in the middle of the SHG image. **(b)** Representative image of retinal layers by H&E staining with the indicated anatomies. OS, IS, and ONL correspond to outer segment, inner segment, and outer nuclear layer, respectively. **(c)** Representative spectra of SHG for image reconstruction (spot (i) in **(a)**). **(d)** Representative retrieved spectra of CARS at the positions of (ii) and (iii) in **(a)**. Right, higher magnification of left at 1800–800 cm^−1^. Because raw CARS spectra were distorted due to interference between vibrationally resonant and non-resonant coherent signals, we used the maximum entropy method (MEM) to retrieve the raw CARS spectra to Im[*χ*^(3)^] spectra corresponding to the conventional spontaneous Raman scattering spectrum. **(e)** Schematic of a photoreceptor highlighting the internal filamentous structures connecting cilia and ciliary rootlets. Parentheses indicate marker antibodies. Bar, 20 μm. Color scales indicate intensity (a.u.) or amplitude (a.u.).

Because the neural retina has a highly organized multilayered structure that is attractive as a model for self-patterning and self-driven morphogenesis^41^, we thoroughly visualized rat retinal layers using SHG (2ω_1_), THG (3ω_1_), and multiplex SFG (ω_1_ + ω_2_), TSFG, and CARS channels of CNOM setup shown in Fig. S2a (Fig. 1 and Fig. S3). The samples were frozen sections of a rat eye. The data were originally obtained as point-by-point spectra at each position within the sample (Fig. 1c,d) and then reconstructed as images (Fig. 1a). Notably, SHG provided high contrast in the upper part of the image (Fig. 1a, left end; SHG). The upper SHG image most likely corresponds to sclera, which is mainly composed of collagen, a well-known harmonophore in living tissue^5^. Nevertheless, the most striking finding was another SHG-positive “filamentous” layer in the middle of the image. The SHG signal intensity from the “filamentous” layer was very weak, which was about 1/1000 of that from sclera. To address the molecular origin of this harmonophore, we compared SHG with other modes of CNOM (Fig. 1a,d). Complementarily, the CARS image at 2860 cm^−1^ (CH_2_ stretch vibrational mode), corresponding to lipids, exhibited high contrast around the outer segment (OS). This most likely corresponds to the distribution of disc membranes in the photoreceptor layer, which is filled with stacks of lipid-rich disc membranes containing the visual pigment molecules^42^. Also, the CARS image at 1570 cm^−1^ (purine ring stretch due to adenine and guanine), corresponding to the distribution of nucleic acid, exhibited high contrast around the nuclei at the outer nuclear layer. These multimodal CNOM images effectively showed the hierarchical structures of retinal layers as merged images (Fig. 1a) and were consistent with the results of hematoxylin–eosin (H&E) staining (Fig. 1b). It should be emphasized that the sample used in Fig. 1a was not H&E stained, but was instead visualized in a label-free manner. Together with the histological aspects, we concluded that this novel SHG reveals filamentous structures that are oriented inside photoreceptor cells; more precisely, these structures are connecting cilia or ciliary rootlets (Fig. 1e).

### Retinal SHG corresponds to photoreceptor ciliary rootlet

**Figure 2:**
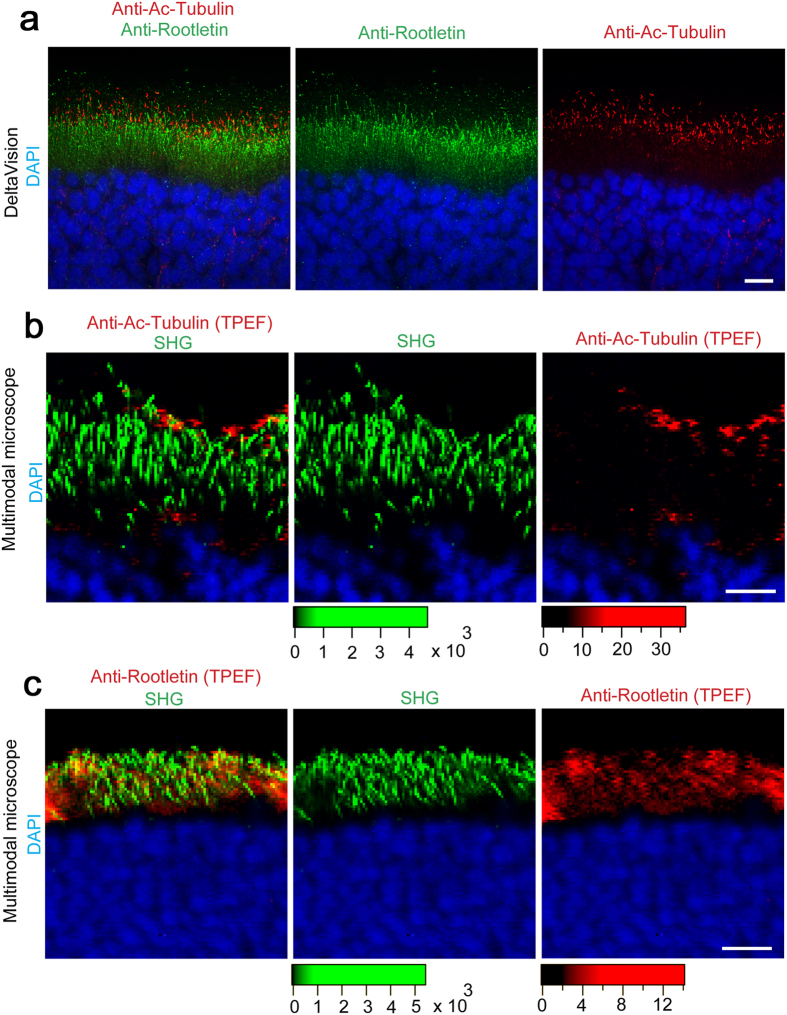
Retinal SHG corresponds to photoreceptor ciliary rootlets. Fixed rat retina was immunostained with the indicated marker antibodies, followed by superimposed observations with DeltaVision as a standard **(a)** or custom-built multimodal microscope equipped for SHG and additional TPEF modes using 775 nm excitation to detect Alexa Fluor 488 immunofluorescence **(b)**. **(a)** Ac-tubulin (red) and Rootletin staining (green) are well separated by standard microscopy, marking two distinct filamentous layers: photoreceptor connecting cilia and ciliary rootlet, respectively. See also the schematic in Fig. 1e. (**b**,**c**) Superimposition of SHG signal (green) and TPEF-detected single marker staining (red). Note that the SHG signal corresponds to the Rootletin staining, indicating ciliary rootlet, but not to ac-tubulin staining, indicating connecting cilia. DAPI (blue) was used as a nuclear marker. Bars, 10 μm. Color scales indicate intensity (a.u.).

To address the origin of the filamentous SHG in retina, we compared it with possible filaments indicated by marker staining using the experimental setup shown in Fig. S2b. Acetylated (ac)-tubulin and Rootletin are markers of connecting cilia and ciliary rootlet, respectively (Figs 1e and 2)^43^. Using standard microscopy, immunostaining with anti-ac-tubulin and anti-Rootletin successfully revealed different layers (Fig. 2a). By utilizing our custom-made nonlinear optical microscope with SHG and two-photon excitation fluorescence (TPEF) modes using 775 nm excitation (Fig. S2b), we found that the SHG is well colocalized with Rootletin, but not with ac-tubulin staining (Fig. 2b,c). Thus, the retinal SHG corresponds to ciliary rootlet.

### SHG is ascribed to the Rootletin R234 domain which assembles into filaments

**Figure 3:**
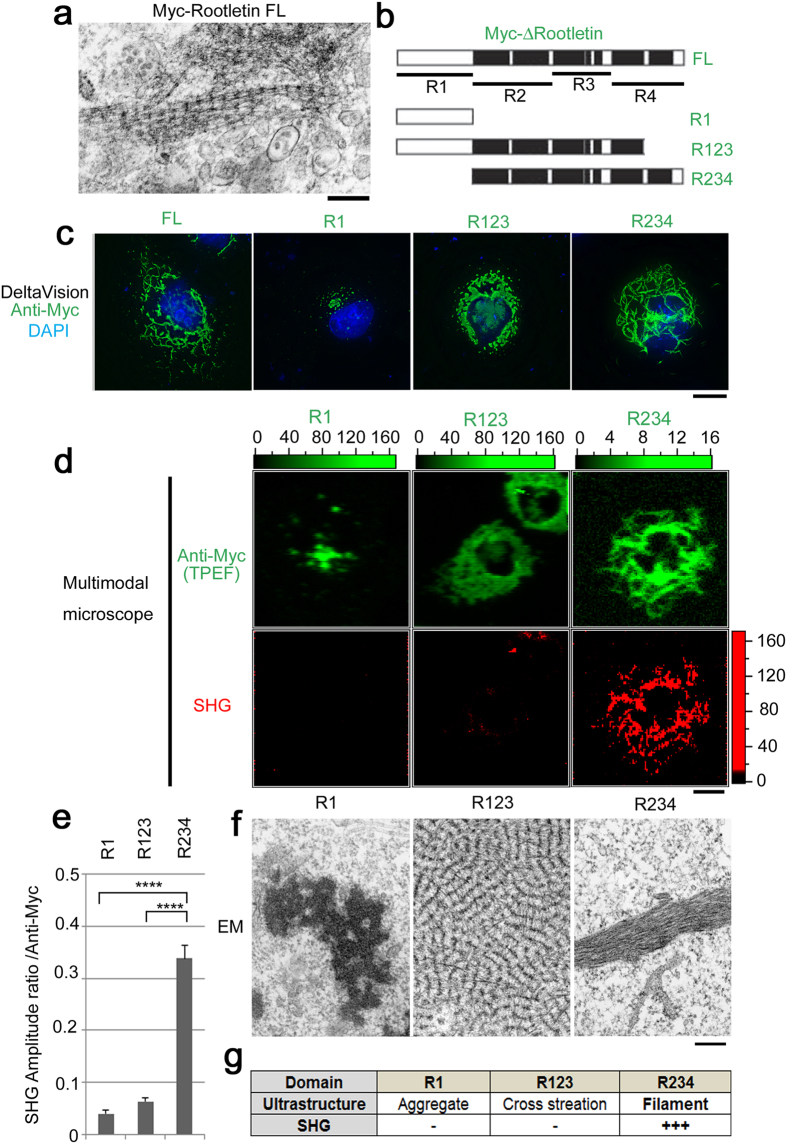
SHG is ascribed to the Rootletin R234 domain, which assembles filaments. Myc-tagged deletion constructs of human Rootletin were introduced into COS7 cells by transfection. Transfectants were fixed and subjected to multiple microscopic observations. **(a)** Full-length Myc-human Rootletin assembles intact filaments with periodic cross-striation at the electron-microscopic level, like endogenous Rootletin filaments. **(b)** Schematic diagram of humanized and Myc-tagged Rootletin deletion mutants: R1, Globular head domain; R2-4, rod domains. See also ref. 35. **(c)** Each human deletion mutant assembles a distinct architecture, as revealed by standard immunofluorescence, as previously reported for the murine case. Note that the minimal filament-forming domain is R234. Anti-Myc (green), DAPI (blue). **(d)** Multimodal microscopy highlights the correlation of SHG with filament assembled from R234 domain. TPEF (green), SHG (red). **(e)** Quantitation of the SHG in (**d**), normalized to Myc intensity per cell. **(f)** Distinct ultrastructures assembled from each fragment: R1, aggregates with high electron density; R123, massive cross-striation; R234, densely packed filaments. **(g)** Summary of multilateral domain analyses. Bars, 200 nm in (**a** and **f**) and 10 μm in (**c** and **d**). Error bars represent s.e.m. ****P < 0.001. *N* = 5 in (**e**). Color scales indicate intensity (a.u.).

To further address the intra-molecular and structural origin of the SHG, we performed domain analysis in transfected cells (Fig. 3). Referring to a previous report in mouse^35^, we introduced a variety of humanized Rootletin fragments expression vectors into cells that built up different structures. Cells expressing full-length Rootletin contained filaments with periodic cross-striation at the electron-microscopic level, in accordance with endogenous Rootletin filaments (Fig. 3a). Similarly, R1, R123, and R234 fragments were also introduced in cells; however, in immunofluorescence analyses, only cells expressing R234 displayed a filamentous pattern similar to that of cells expressing the full-length protein (Fig. 3b,c). When we compared TPEF and SHG using our custom microscope, we found that the SHG was prominent on filaments assembled from R234, but not on aggregates formed from R1 or R123 (Fig. 3d,e). Since the SHG intensity (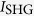) shows quadratic dependence with respect to the amount of molecular emitters within the focal volume, we used the ratio of the SHG amplitude (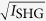) to the TPEF intensity of anti-Myc to analyze data quantitatively as shown in Fig. 3e.

These results are possibly correlated to the molecular structure of Rootletin. In our electron microscopic data, overexpressed R123 and R234 corresponded to different structures: cross-striation and filament, respectively (Fig. 3f). According to the literature^35^, R123 is composed of two domains, (1) globular R1 domain that does not contribute to the formation of filaments and (2) R23 as a partially deleted rod domain; R234 is devoid of the globular R1 domain but retaining the entire rod domain that corresponds to filament formation. Therefore, we consider that the supra-molecular organization of the R123 fragment, indicated as cross-striation, is mainly due to globular R1 domain and is different from filaments assembled from R234 entire rod domain, which gives rise to different SHG intensity. In more detail: R1 aggregate was SHG-negative, and R123 cross-striation also made little contribution to the SHG. By contrast, only longitudinal R234 filaments contribute to SHG. Consequently, we could attribute the SHG of Rootletin to the filamentous ultrastructure assembled from the R234 domain (Fig. 3g).

### SHG induction shows quantitative agreement with the density of R234 filaments

**Figure 4:**
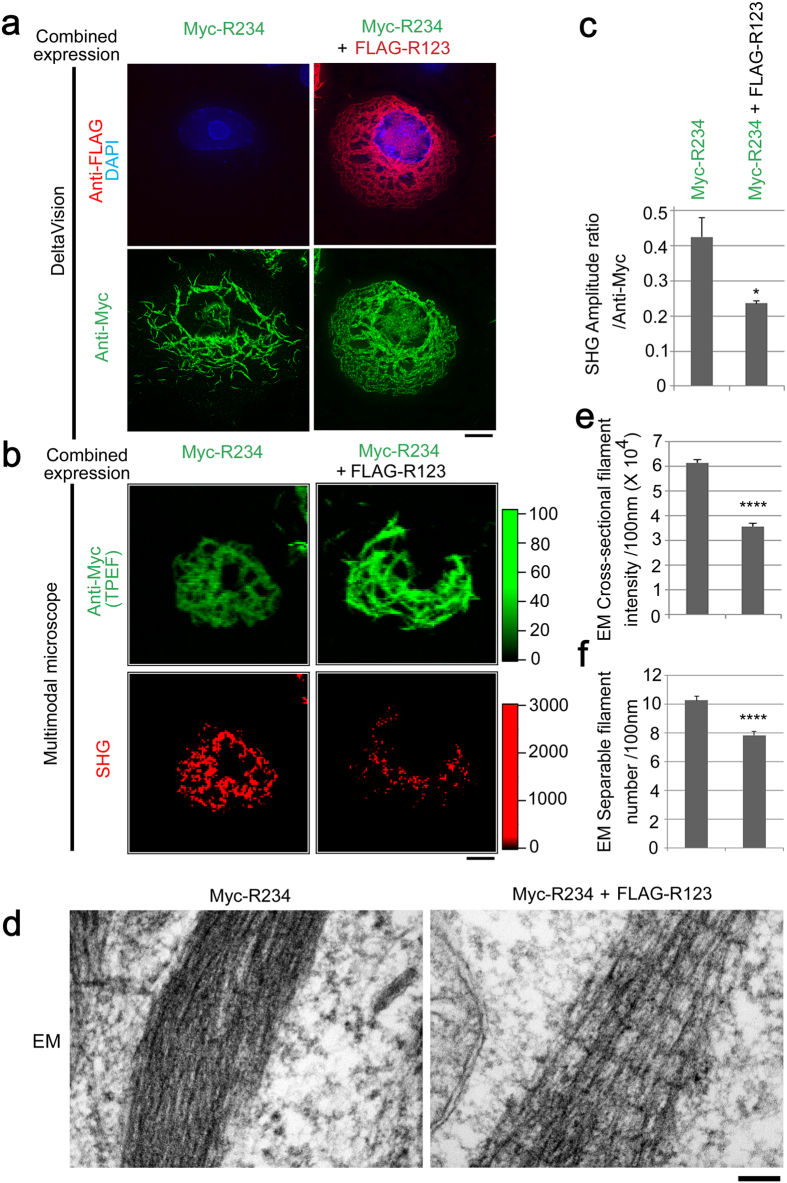
SHG induction shows quantitative agreement with the density of R234 filaments. **(a)** Standard immunofluorescence highlighted unsharpened Myc-R234 filaments co-expressed with the FLAG-R123 domain. Anti-Flag (red), Anti-Myc (green), DAPI (blue). **(b)** SHG induction was weaker under co-expression. TPEF (green), SHG (red). **(c)** Quantitative reduction of SHG in (**b**), compared to Myc intensity in each cell. **(d)** Electron micrograph depicting ultrastructural alteration due to FLAG-R123 co-expression: (left) pure R234 formed packed filaments; (right) FLAG-R123 co-expression resulted in formation of more widely spaced filaments with additional cross-striations, similar to the case of full-length Rootletin (Fig. 3a). (**e** and **f**) Quantitation of filaments in (**d**), as both intensity (**e**) and number (**f**) note the correlation to SHG in **c**. Bars, 10 μm in (**a** and **b**), and 100 nm in (**d**). Error bars represent s.e.m. *P < 0.05 in (**c**), ****P < 0.001 in (**e** and **f**). *N* = 5, 30, and > 28 in (**c**,**e** and **f**), respectively. Color scales indicate intensity (a.u.).

Next, we performed the quantitative assessment of the R234-induced SHG (Fig. 4). We utilized the R123 fragment as a dominant-negative mutant for filament assembly, as described in a previous report^35^. As expected, Myc-R234–assembled filaments became blurred by combined expression with FLAG-R123 fragments (Fig. 4a). Using our custom microscope (Fig. S2b), we also found that SHG was decreased by the co-expression, compared to the amount of Myc-R234 expression (Fig. 4b,c). Electron microscopy revealed that Myc-R234 filaments containing FLAG-R123 became sparser than Myc-R234 assembled alone, with respect to both number and intensity (Fig. 4d–f). Combined expression restored closely spaced Rootletin filaments like those shown in Fig. 2a, assembling filaments with cross-striation possibly originating from the R123 domain, indicating mutual complementation. It should be noticed that the changes of molecular organization and alignment of Myc-R234 filaments due to FLAG-R123 can give rise to strong difference in the detected SHG signal intensity, even if the structural change is small. It means that SHG can be used as a sensitive probe to investigate structural changes in the scale of sub-micrometer and micrometer. Based on these quantitative assessments, we conclude that the SHG can be ascribed to a filamentous ultrastructure assembled from R234 domains, but not to unassembled monomeric R234 itself. Complete assignment and determination of the filament diameter could be performed by absolute measurement of SHG signals^44^.

### Cellular SHG is derived from Rootletin filaments

**Figure 5:**
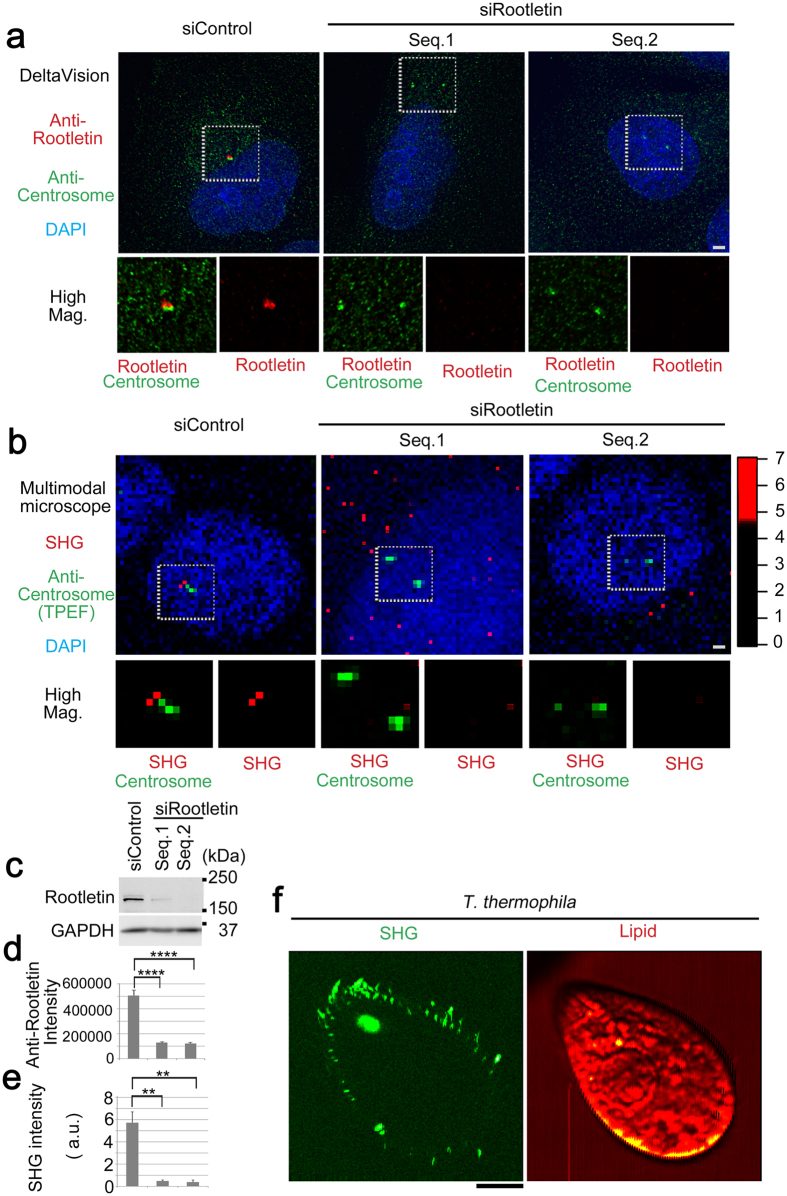
Cellular SHG is derived from Rootletin filaments. **(a–e)** U2OS cells were treated with negative control or Rootletin-specific siRNA, followed by immunofluorescence for multimodal observations. **(a)** Standard double immunofluorescence confirmed both Rootletin staining proximal to the centrosome (left) and its depletion by siRootletin, accompanied by centrosome splitting (middle and right). Anti-Rootletin (green), SHG (red). **(b)** Multimodal microscopic observation revealed SHG induction in Rootletin-positive adjacent centrosomes (left) but not in Rootletin-depleted split centrosomes (middle and right). TPEF (green), SHG (red). **(c)** Immunoblot confirming Rootletin knockdown. (**d** and **e**) Quantitative data for (**a** and **b**), respectively; note the mutual correlation. **(f)** Multimodal images of *T. thermophila* as xy plane; note a lot of fibrous signals in SHG (green). Lipid (red and yellow) as a control. Pericentrin 2 or γ-tubulin was used as a centrosomal marker. DAPI (blue) was used as a nuclear marker. Bars, 2 μm. GAPDH was used as a loading control. Error bars represent s.e.m. ****P < 0.001 and **P < 0.01 in d and e, respectively. *N* = 10 and 5 in (**d** and **e**), respectively. Color scales indicate intensity (a.u.).

Finally, we evaluated the Rootletin-induced SHG in cultured cells. Rootletin forms centriole-associated fibrous structures that contribute to the establishment of centrosome cohesion before mitosis^24^. Consistent with this, knockdown of Rootletin resulted in centrosome dissociation, accompanied by the loss of Rootletin signals (Fig. 5a,c). Next, we examined the specimen under our custom microscope, with centrosome marking (Fig. 5b). In control cells, a distinct SHG was observed in the vicinity of unseparated centrosomes; however, in Rootletin-depleted cells, this SHG disappeared from the phenotypic split centrosome (Fig. 5b,d,e). Thus, we concluded that the centrosomal SHG is most likely derived from Rootletin filaments involved in centrosome cohesion.

Then, we again challenged to expand this utility of SHG-specificity toward unicellular ciliates. Fortunately, we found that a lot of fibrous SHG were observed even in *Tetrahymena thermophila*, most likely indicating kinetodesmal fibers as an analogous to Rootletin filaments (ref. 45, Fig. 5f). Thus, it is convincing that SHG-specificity of cellular Rootletin filaments enables naïve imaging with universal conservation.

## Discussion

In this study, we discovered distinct SHG that detects Rootletin filaments in tissues and cultured cells. This concept can be expanded to unicellular ciliates. This conclusion was reached not only by observing endogenous Rootletin (Fig. 2) but also by introducing exogenous truncated mutants, demonstrating that filaments assembled from the Rootletin R234 domain are the ultrastructure responsible for the SHG (Figs 3 and 4). Also, knockdown cells confirmed that Rootletin was specific to the centrosomal SHG (Fig. 5). Besides, due to characteristic of SHG imaging and Rootletin itself, following items should be discussed.

We investigated the assembled structures of filaments by polarization SHG microscopy using harmonophores as probes. Figure S4a shows the SHG image of retinal layers and its polarization dependence. We analyzed the SHG signal of collagen fibrils in sclera (top) and Rootletin filaments in photoreceptor cells (middle). The SHG signal intensity was strongly dependent on the angle (*α*) between the incident laser polarization and the orientation of the filamentous structures. The results (Fig. S4b) show that the polarization dependences of collagen and Rootletin clearly differed. Collagen gave an SHG signal maximum around 0 and 180 degrees, consistent with a previous report^46^. On the other hand, Rootletin gave a maximum around 45 and 135 degrees, similar to that of myosin^46^. Since Rootletin is close to myosin in the phylogenetic tree^35^ from public protein databases, this result could be reasonable. It implies that Rootletin filaments could form assembled structures similar to myosin, i.e., tail–tail structures.

Myc-tagging enabled definition of Rootletin-containing filamentous unit. In a previous study using the R234 domain, the GFP tag potentially compensated for the R1 globular domain, enabling assembly into intact Rootletin filaments with cross striations^35^. In our case, Myc-tagged R234 assembled packed filaments that had no cross-striations (Fig. 3f) and were sensitive to forced spacing by FLAG-R123 cross-striations, which enabled a quantitative assessment (Fig. 4). As shown in Fig. 4b, perturbed Myc-R234 filament formation exhibited less SHG intensity than expected from the Myc intensity. This indicates that a great deal of Myc-R234 monomer that does not form filaments is released by this perturbation. Since the SHG signal is boosted through coherent buildup inside filaments, the signal intensity is sensitive to the molecular organization and alignment of filaments. Consequently, the SHG most likely originated from the ultrastructurally recognized filamentous structure, but not from free R234 monomers.

Clinically, degeneration of ciliary rootlets over time is closely associated with visual loss and impaired mucociliary clearance in the trachea^36^. Although the laser power should be minimized in the future, such label-free observation of retinal ciliary rootlets has potential, especially for prognosis of late-life blindness; in such patients, quality of life (QOL) should be carefully considered.

In cell biology, Rootletin has a conserved function mediating ciliary chemo- and mechano-sensing^36^,^37^,^38^. In such research field, exogenous labeling often perturbs endogenous function, but SHG can surpass such potential artifacts. By increasing sensitivity or decreasing exposure time it may be possible to achieve live imaging.

Furthermore, we found that analogous structures of Rootletin filaments were also SHG-active even in unicellular ciliates (Fig. 5f). Because the proteic components of unicellular Rootletin filaments have low sequence homology to those of mammalian ones^35^,^45^, it is intriguing that the distinct SHG-activity depend on rather higher order structure than sequence homology itself.

By expanding this structural concept, SHG may act as a novel probe for sensors based on their steric properties and thus has the potential to overcome a major shortcoming of current proteomics, which relies on protein–protein interactions, or genomics, which is based on one-dimensional sequence similarities. Given that we and others have detected additional SHG signals during other specific cellular events and in other organisms^47^, many SHG-active cellular components and the responding proteins remain to be determined, including possible mechano-mediators such as Rootletin. Of course, the combination of polarization dependence and multimodal CNOM will contribute to higher molecular definition. We are currently embarking on a pioneering era in next-generation photonics, as applied to histology, molecular cell biology, or cellular engineering, for which the major challenge is the development of label-free observable substances in multiple organisms.

## Methods

### Custom-made penta-modal coherent nonlinear optical microscopic (CNOM) system

A schematic of our custom-made CNOM^16^ is shown in Fig. S2a. Owing to recent advances in supercontinuum (SC) generation technology, our CNOM system has extended to ultra-broadband spectroscopic live cell imaging^16^. The master laser source was a microchip Nd:YAG laser, which is a simple, low-cost, robust, and compact source. The wavelength, temporal duration, repetition rate, and output average power were 1064 nm, 0.8 ns, 33 kHz, and 300 mW, respectively. The output from the laser source was divided in two: one portion was used directly as the pump (ω_1_) beam, and the other was introduced into a photonic crystal fiber (PCF) to generate SC with a spectral range from visible to NIR. The laser source and PCF are assembled and packed as an integrated SC laser source (Leukos, Limoges, France). After passing through several NIR-pass filters, only NIR spectral components remained; these were used as the Stokes (ω_2_) beam. Both the ω_1_ and ω_2_ beams were superposed and introduced into the modified microscope (ECLIPSE Ti-U; Nikon, Tokyo, Japan). The ω_1_ and ω_2_ beams were tightly focused onto the sample using a water-immersion microscope objective (CFI Plan Apo 60x NA 1.27; Nikon, Tokyo, Japan), giving rise to a signal due to nonlinear optical processes. The laser power density at the sample position was about 10 MW/cm^2^ both for ω_1_ and ω_2_ laser. The sample was scanned to obtain images using a piezo stage (LP-200; MadCityLabs, Madison, WI). Owing to the broadband spectral profile of the ω_2_ beam, we could obtain a multiplex CARS signal (2ω_1_ − ω_2_) simultaneously for all fundamental vibrational modes in the NIR region. Moreover, the ω_1_ and ω_2_ beams also generated other nonlinear optical processes such as SHG (2ω_1_), SFG (ω_1_ + ω_2_), THG (3ω_1_), and TSFG (2ω_1_ + ω_2_ and ω_1_ + 2ω_2_), which ranged from ultraviolet (UV) to the visible region (See also Fig. S1). It should be noted that all signals were acquired simultaneously using a cost-effective sub-nanosecond laser source^16^,^48^. The signals from the sample were collimated by another microscope objective (Plan S Fluor 40x NA 0.6; Nikon, Tokyo, Japan) and divided by a dichroic mirror in such a manner that the UV-visible and NIR signals were separately detected by two sets of spectrometers (polychromator (LS785; Princeton Instruments, Princeton, NJ) equipped with a CCD camera (PIXIS-BR; Princeton Instruments, Princeton, NJ) and polychromator (Spectra-Pro320i; Princeton Instruments, Princeton, NJ) equipped with a CCD camera (PIXIS-B; Princeton Instruments, Princeton, NJ) for NIR and UV-visible, respectively). In this study, *penta*-modal (CARS, SHG, SFG, THG, and TSFG) label-free imaging was performed using the sub-nanosecond SC source. The exposure time for each pixel was 50 ms, and the total image acquisition time was about 34 min for the images with 201 × 201 pixels (Fig. 1). The pixel dwell time in the present study is much longer than that of the typical SHG microscopic setup using the high repetition-rate laser source (a few μs). In fact, that is the very point that we were able to detect the very weak SHG signal from Rootletin. As shown in Fig. S3, the signal intensity is about 1/1000 of that from collagen in sclera. Such a long exposure time is required to obtain the CARS signal with ultrabroad spectral information. Although the total measurement time for image acquisition is 34 min (Fig. 1) and 85 min (Fig. 2b,c), our setup is robust and stable enough to obtain multimodal images with high image contrast. The focal volume is about 0.5 μm and 2–3 μm in the lateral and axial directions, respectively. The focal volume is large enough to sum up the coherent signal inside the Rootletin filament, whose diameter is typically in the order of 100 nm.

### Bi-modal (SHG and TPEF) imaging by 775 nm excitation

Because the excitation wavelength of the original custom-made penta-modal CNOM was ≥1064 nm, we could not excite typical fluorophores such as Alexa Fluor 488. In order to achieve simultaneous measurement of SHG and TPEF signals, we incorporated another pulsed laser source (KATANA LP; Onefive GmbH, Regensdorf, Switzerland) as the ω_3_ laser into the existing penta-modal CNOM (Fig. S2b). Typical wavelength, temporal duration, repetition rate, and output averaged power were 775 nm, 50 ps, 100 kHz, and 75 mW, respectively. As shown in Fig. S2b, the dichroic mirror in the microscope was just replaced to introduce the 775 nm laser pulses. The laser power density at the sample position was about 1 MW/cm^2^. The exposure time for each pixel was 500 ms, and the total image acquisition time was about 85 min (101 × 101 pixels).

Figure S5 shows a spectral profile of immunostained retina at the position of the SHG filament with 775 nm laser excitation. Two bands were observed: a sharp and intense band at 388 nm, and a broad and weak band around 520 nm with three peaks (440, 475, and 550 nm), corresponding to SHG and TPEF, respectively. The bands around 440 and 520 nm were assigned as TPEF due to DAPI and Alexa Fluor 488, respectively, with which the tissue had been stained. On the other hand, the band around 475 nm was also observed in a non-stained sample. Therefore, it was assigned as auto-fluorescence. The existence of autofluorescence was also confirmed using a hyper-spectral fluorescence imaging system (Nuance; PerkinElmer, Waltham, MA). It should be noted that the autofluorescence was prominent only for tissue samples; it was weak or almost negligible for cultured cells. We first decomposed the spectral profile of the TPEF signal into three bands corresponding to DAPI, Alexa Fluor 488, and autofluorescence, and then reconstructed the TPEF images of DAPI and Alexa 488. It should be emphasized that such detailed spectral decomposition can be performed using spectroscopic detection.

### Cell culture and transfection

COS7 and U2OS cells (ATCC, Manassas, VA) were cultured in DMEM containing 10% fetal bovine serum. They were authenticated by species-specific PCR^49^ or by short tandem repeat (STR) profiling techniques, respectively. All cell lines were free from mycoplasma contamination, confirmed by TaKaRa PCR Mycoplasma Detection Set (TaKaRa, Shiga, Japan). Transfection of plasmids or siRNAs transfection was performed using ViaFect (Promega, Madison, WI) or Lipofectamine RNAiMAX (Life Technologies, Grand Island, NY), respectively. *Tetrahymena thermophila* was maintained as a previous report^50^.

### DNA constructs and siRNAs

Myc-tagged full-length human Rootletin was a gift from Erich Nigg (Addgene plasmid # 41167)^24^. In a previous study, R1, R123, and R234-deletion constructs were generated from mouse Rootletin and GFP-tagged^35^; these constructs were thoroughly humanized, and the GFP tag was replaced with Myc or FLAG to minimize side effects. Rootletin was depleted using siRNAs targeting the following sequences (Life Technologies, Grand Island, NY): sequence 1 (Sq. 1), 5′-GGGAGATTGTCACCCGCAA-3′; Sq. 2, 5′-GGCCTGCGGCAGCAAATAA-3′. Silencer Select negative control #2 siRNA (Life Technologies, Grand Island, NY) was used as a negative control.

### Antibodies

Antibodies were obtained from the indicated suppliers: goat anti-Rootletin for immunostaining (sc-67824) and mouse anti-Rootletin for immunoblotting (sc-374056; Santa Cruz Biotechnology, CA); mouse anti-acetylated tubulin (6–11B-1), mouse anti-FLAG (M2), and mouse anti-gamma-tubulin (T3559; Sigma-Aldrich, St. Louis, MO); mouse anti-Myc (9B11; Cell Signaling Technology, Danvers, MA); and rabbit anti-Pericentrin 2 (ab4448) and HRP-conjugated anti-glyceraldehyde 3-phosphate dehydrogenase (GAPDH, ab9385; Abcam, MA). Species-specific secondary antibodies were conjugated to Alexa Fluor 488 or 555 (Life Technologies, Grand Island, NY). Western blot analyses were performed as described previously^31^.

### Fixation, immunostaining, and standard fluorescence microscopy

F344/NSlc rats were purchased from Japan SLC, Inc. (Hamamatsu, Japan). All animal experiments followed the Association for Research in Vision and Ophthalmology guidelines and were approved by the Animal Care and Use Committees of University of Tsukuba and Aichi Cancer Center Research Institute. A Rat eye was frozen in liquid nitrogen and cut at 20 μm on a cryostat, mounted on coverslips (Iwaki Glass Co., Ltd. Tokyo, Japan), air dried, and fixed in 1% formaldehyde for 15 min. Cultured cells were grown on coverslips and similarly fixed, followed by permeabilization with PBS containing 0.2% Triton X-100 for 15 min. For immunostaining, they were blocked with 1% BSA/PBS for more than 15 min and incubated with primary antibodies for 1 h. After being washed with PBS three times, samples were incubated for 30 min with secondary antibodies. DeltaVision system (GE Healthcare, Seattle, WA) was used for standard fluorescence images, equipped with a microscope (IX70; Olympus, Tokyo, Japan), Plan Apochromat 60×/1.42 and 100×/1.40 NA oil immersion lenses (Olympus, Tokyo, Japan), and a CCD camera (CoolSNAP HQ; Photometrics, Tucson, AZ). The images were obtained in Z sections at 0.2 μm intervals, deconvoluted, and integrated using the softWoRx software (GE Healthcare, Seattle, WA). Images were taken at room temperature and further processed using Photoshop Elements 6.0 (Adobe, San Jose, CA) according to the *Scientific Reports* guidelines. Conventional hematoxylin and eosin (H&E) staining was performed as described previously^51^.

### Transmission electron microscopy

COS7 cells cultured on coverslips were fixed with 2% fresh formaldehyde and 2.5% glutaraldehyde in 0.1 M sodium cacodylate buffer, pH 7.4, for 2 h at room temperature. After washing with 0.1 M cacodylate buffer, pH 7.4, they were postfixed with ice-cold 1% OsO_4_ in the same buffer for 2 h. The samples were rinsed with distilled water, stained with 0.5% aqueous uranyl acetate for 2 h or overnight at room temperature, dehydrated with ethanol and propylene oxide, and embedded in Polybed 812 (PolySciences, Inc., Warrington, PA). After removal of coverslips using ice-cold hydrofluoric acid, ultra-thin sections were cut, doubly stained with uranyl acetate and Reynolds’s lead citrate, and viewed with a transmission electron microscope equipped with a charge-coupled device camera (JEM-1400 plus; JEOL, Tokyo, Japan).

### Statistical analyses

Results are presented as mean plus s.e.m., as specified in each figure legend. Significance was calculated by double-tailed t-test using Excel (Microsoft, Redmond, WA). *P < 0.05, **P < 0.01, ***P < 0.005, and ****P < 0.001 were considered as statistically significant differences.

## Additional Information

**How to cite this article**: Akiyama, T. *et al*. SHG-specificity of cellular Rootletin filaments enables naïve imaging with universal conservation. *Sci. Rep.* **7**, 39967; doi: 10.1038/srep39967 (2017).

**Publisher's note:** Springer Nature remains neutral with regard to jurisdictional claims in published maps and institutional affiliations.

## Supplementary Material

Supplementary Information

## References

[b1] Min W., Freudiger C. W., Lu S. J. & Xie X. S. Coherent Nonlinear Optical Imaging: Beyond Fluorescence Microscopy. Annu. Rev. Phys. Chem. 62, 507–530 (2011). 21453061 10.1146/annurev.physchem.012809.103512 PMC3427791

[b2] Muller M. & Schins J. M. Imaging the thermodynamic state of lipid membranes with multiplex CARS microscopy. J. Phys. Chem. B 106, 3715–3723 (2002).

[b3] Petrov G. I. . Comparison of coherent and spontaneous Raman microspectroscopies for noninvasive detection of single bacterial endospores. Proc. Natl. Acad. Sci. USA 104, 7776–7779 (2007). 17483468 10.1073/pnas.0702107104 PMC1876523

[b4] Moreaux L., Sandre O., Charpak S., Blanchard-Desce M. & Mertz J. Coherent scattering in multi-harmonic light microscopy. Biophys. J. 80, 1568–1574 (2001). 11222317 10.1016/S0006-3495(01)76129-2 PMC1301348

[b5] Campagnola P. J. . Three-dimensional high-resolution second-harmonic generation imaging of endogenous structural proteins in biological tissues. Biophys. J. 82, 493–508 (2002). 11751336 10.1016/S0006-3495(02)75414-3 PMC1302489

[b6] Matteini,. P. . Thermal transitions of fibrillar collagen unveiled by second-harmonic generation microscopy of corneal stroma. Biophys. J. 103, 1179–1187 (2012). 22995490 10.1016/j.bpj.2012.07.055 PMC3446693

[b7] Pavone F. S. & Campagnola P. J. Second Harmonic Generation Imaging. (CRC Press, 2013).

[b8] Cicchi R. . *In vivo* non-invasive monitoring of collagen remodelling by two-photon microscopy after micro-ablative fractional laser resurfacing. Journal of biophotonics 7, 914–925 (2014). 24339127 10.1002/jbio.201300124

[b9] Gauderon R., Lukins P. B. & Sheppard C. J. R. Optimization of second-harmonic generation microscopy. Micron 32, 691–700 (2001). 11334739 10.1016/s0968-4328(00)00066-4

[b10] Zumbusch A., Holtom G. R. & Xie X. S. Three-dimensional vibrational imaging by coherent anti-Stokes Raman scattering. Phys. Rev. Lett. 82, 4142–4145 (1999).

[b11] Hashimoto M., Araki T. & Kawata S. Molecular vibration imaging in the fingerprint region by use of coherent anti-Stokes Raman scattering microscopy with a collinear configuration. Opt. Lett. 25, 1768–1770 (2000). 18066338 10.1364/ol.25.001768

[b12] Freudiger C. W. . Label-Free Biomedical Imaging with High Sensitivity by Stimulated Raman Scattering Microscopy. Science 322, 1857–1861 (2008). 19095943 10.1126/science.1165758 PMC3576036

[b13] Ozeki Y. . High-speed molecular spectral imaging of tissue with stimulated Raman scattering. Nature Photon. 6, 845–851 (2012).

[b14] Ranka J. K., Windeler R. S. & Stentz A. J. Visible continuum generation in air-silica microstructure optical fibers with anomalous dispersion at 800 nm. Opt. Lett. 25, 25–27 (2000). 18059770 10.1364/ol.25.000025

[b15] Raghunathan V., Han Y., Korth O., Ge N.-H. & Potma E. O. Rapid vibrational imaging with sum frequency generation microscopy. Opt. Lett. 36, 3891–3893 (2011). 21964132 10.1364/OL.36.003891 PMC4157057

[b16] Segawa H. . Label-free tetra-modal molecular imaging of living cells with CARS, SHG, THG and TSFG (coherent anti-Stokes Raman scattering, second harmonic generation, third harmonic generation and third-order sum frequency generation). Opt. Express 20, 9551–9557 (2012). 22535046 10.1364/OE.20.009551

[b17] Freund I., Deutsch M. & Sprecher A. Connective tissue polarity. Optical second-harmonic microscopy, crossed-beam summation, and small-angle scattering in rat-tail tendon. Biophys. J. 50, 693–712 (1986). 3779007 10.1016/S0006-3495(86)83510-X PMC1329848

[b18] Plotnikov S. V., Millard A. C., Campagnola P. J. & Mohler W. A. Characterization of the myosin-based source for second-harmonic generation from muscle sarcomeres. Biophys. J. 90, 693–703 (2006). 16258040 10.1529/biophysj.105.071555 PMC1367074

[b19] Dombeck D. A. . Uniform polarity microtubule assemblies imaged in native brain tissue by second-harmonic generation microscopy. Proc. Natl. Acad. Sci. USA 100, 7081–7086 (2003). 12766225 10.1073/pnas.0731953100 PMC165833

[b20] Singla V. & Reiter J. F. The primary cilium as the cell’s antenna: signaling at a sensory organelle. Science 313, 629–633 (2006). 16888132 10.1126/science.1124534

[b21] Ishikawa H. & Marshall W. F. Ciliogenesis: building the cell’s antenna. Nat. Rev. Mol. Cell Biol. 12, 222–234 (2011). 21427764 10.1038/nrm3085

[b22] Gerdes J. M., Davis E. E. & Katsanis N. The Vertebrate Primary Cilium in Development, Homeostasis, and Disease. Cell 137, 32–45 (2009). 19345185 10.1016/j.cell.2009.03.023 PMC3016012

[b23] Fliegauf M., Benzing T. & Omran H. When cilia go bad: cilia defects and ciliopathies. Nat. Rev. Mol. Cell Biol. 8, 880–893 (2007). 17955020 10.1038/nrm2278

[b24] Bahe S., Stierhof Y. D., Wilkinson C. J., Leiss F. & Nigg E. A. Rootletin forms centriole-associated filaments and functions in centrosome cohesion. J. Cell Biol. 171, 27–33 (2005). 16203858 10.1083/jcb.200504107 PMC2171225

[b25] Hildebrandt F., Benzing T. & Katsanis N. Mechanisms of Disease: Ciliopathies. New Engl. J. Med. 364, 1533–1543 (2011). 21506742 10.1056/NEJMra1010172 PMC3640822

[b26] Goetz S. C. & Anderson K. V. The primary cilium: a signalling centre during vertebrate development. Nature Reviews Genetics 11, 331–344 (2010). 10.1038/nrg2774 PMC3121168 20395968

[b27] Bloodgood R. A. From central to rudimentary to primary: the history of an underappreciated organelle whose time has come. The primary cilium. Methods Cell Biol. 94, 2–52 (2009). 10.1016/S0091-679X(08)94001-2 20362083

[b28] Kobayashi T. & Dynlacht B. D. Regulating the transition from centriole to basal body. J. Cell Biol. 193, 435–444 (2011). 21536747 10.1083/jcb.201101005 PMC3087006

[b29] Kim S. & Dynlacht B. D. Assembling a primary cilium. Curr. Opin. Cell Biol. 25, 506–511 (2013). 23747070 10.1016/j.ceb.2013.04.011 PMC3729615

[b30] Tucker R. W., Pardee A. B. & Fujiwara K. Centriole ciliation is related to quiescence and DNA synthesis in 3T3 cells. Cell 17, 527–535 (1979). 476831 10.1016/0092-8674(79)90261-7

[b31] Inoko A. . Trichoplein and Aurora A block aberrant primary cilia assembly in proliferating cells. J. Cell Biol. 197, 391–405 (2012). 22529102 10.1083/jcb.201106101 PMC3341160

[b32] Goto H., Inoko A. & Inagaki M. Cell cycle progression by the repression of primary cilia formation in proliferating cells. Cell. Mol. Life Sci. 70, 3893–3905 (2013). 23475109 10.1007/s00018-013-1302-8 PMC3781298

[b33] Engelmann T. W. Zur anatomie und physiologie der flimmerzellen. Pfluegers Arch./Eur. J. Physiol. 23, 505–535 (1880).

[b34] Fawcett D. W. & Porter K. R. A Study of the Fine Structure of Ciliated Epithelia. J. Morphol. 94, 221–282 (1954).

[b35] Yang J. . Rootletin, a novel coiled-coil protein, is a structural component of the ciliary rootlet. J. Cell Biol. 159, 431–440 (2002). 12427867 10.1083/jcb.200207153 PMC2173070

[b36] Yang J. . The ciliary rootlet maintains long-term stability of sensory cilia. Mol. Cell. Biol. 25, 4129–4137 (2005). 15870283 10.1128/MCB.25.10.4129-4137.2005 PMC1087714

[b37] Mohan S., Timbers T. A., Kennedy J., Blacque O. E. & Leroux M. R. Striated Rootlet and Nonfilamentous Forms of Rootletin Maintain Ciliary Function. Curr. Biol. 23, 2016–2022 (2013). 24094853 10.1016/j.cub.2013.08.033

[b38] Chen J. V. . Rootletin organizes the ciliary rootlet to achieve neuron sensory function in Drosophila. J. Cell Biol. 211, 435–453 (2015). 26483560 10.1083/jcb.201502032 PMC4621839

[b39] Styczynska-Soczka K. & Jarman A. P. The Drosophila homologue of Rootletin is required for mechanosensory function and ciliary rootlet formation in chordotonal sensory neurons. Cilia 4 (2015). 10.1186/s13630-015-0018-9 PMC4489026 26140210

[b40] Andersen J. S. . Proteomic characterization of the human centrosome by protein correlation profiling. Nature 426, 570–574 (2003). 14654843 10.1038/nature02166

[b41] Sasai Y. Cytosystems dynamics in self-organization of tissue architecture. Nature 493, 318–326 (2013). 23325214 10.1038/nature11859

[b42] Boesze-Battaglia K. & Schimmel R. J. Cell membrane lipid composition and distribution: Implications for cell function and lessons learned from photoreceptors and platelets. J. Exp. Biol. 200, 2927–2936 (1997). 9359876 10.1242/jeb.200.23.2927

[b43] Rachel R. A. . CEP290 alleles in mice disrupt tissue-specific cilia biogenesis and recapitulate features of syndromic ciliopathies. Hum. Mol. Genet. 24, 3775–3791 (2015). 25859007 10.1093/hmg/ddv123 PMC4459394

[b44] Bancelin S. . Determination of collagen fibril size via absolute measurements of second-harmonic generation signals. Nat. Commun. 5, 4920 (2014). 25223385 10.1038/ncomms5920

[b45] Galati D. F. . DisAp-dependent striated fiber elongation is required to organize ciliary arrays. J. Cell Biol. 207, 705–715 (2014). 25533842 10.1083/jcb.201409123 PMC4274257

[b46] Tiaho F., Recher G. & Rouede D. Estimation of helical angles of myosin and collagen by second harmonic generation imaging microscopy. Opt. Express 15, 12286–12295 (2007). 19547597 10.1364/oe.15.012286

[b47] Ishitsuka K. . Identification of intracellular squalene in living algae,Aurantiochytrium mangroveiwith hyper-spectral coherent anti-Stokes Raman microscopy using a sub-nanosecond supercontinuum laser source. J. Raman Spectrosc. (2016).

[b48] Lefort C. . Multicolor multiphoton microscopy based on a nanosecond supercontinuum laser source. J. Biophotonics, 1–6 (2016). 10.1002/jbio.201500283 26872004

[b49] Ono K. . Species identification of animal cells by nested PCR targeted to mitochondrial DNA. In Vitro Cell. Dev. Biol. Anim. 43, 168–175 (2007). 17516125 10.1007/s11626-007-9033-5

[b50] Kushida Y., Nakano K. & Numata O. Amitosis requires γ-tubulin-mediated microtubule assembly in Tetrahymena thermophila. Cytoskeleton 68, 89–96 (2011). 21246753 10.1002/cm.20496

[b51] Tanaka H. . Cytokinetic Failure-induced Tetraploidy Develops into Aneuploidy, Triggering Skin Aging in Phosphovimentin-deficient Mice. J. Biol. Chem. 290, 12984–12998 (2015). 25847236 10.1074/jbc.M114.633891 PMC4505553

