# Supplementary material for: SHG-specificity of cellular Rootletin filaments enables naïve imaging with universal conservation
Source: Sci Rep. 2017 Jan 6;7:39967. doi: 10.1038/srep39967 (PMC5216331; doi:10.1038/srep39967)
Supplement: Supplementary Information [file srep39967-s1.pdf]

SUPPLEMENTARY INFORMATION

SHG-specificity of cellular Rootletin filaments enables naïve imaging with universal conservation

Toshihiro Akiyama[1¶], Akihito Inoko[2*¶], Yuichi Kaji[3], Shigenobu Yonemura[4,5], Kisa Kakiguchi[4], Hiroki Segawa[6†], Kei Ishitsuka[1], Masaki Yoshida[7], Osamu Numata[7], Philippe Leproux[8], Vincent Couderc[8], Tetsuro Oshika[3], Hideaki Kano[1,9,10*]

[1]*Department of Applied Physics, Graduate School of Pure and Applied Sciences, University of Tsukuba, 1-1-1 Tennodai, Tsukuba, Ibaraki 305-8573, Japan*

[2]*Division of Biochemistry, Aichi Cancer Center Research Institute, 1-1 Kanokoden, Chikusa-ku, Nagoya, Aichi 464-8681, Japan*

[3] *Department of Ophthalmology, Faculty of Medicine, University of Tsukuba, 1-1-1 Tennodai, Tsukuba, Ibaraki 305-8575, Japan*

[4] *Ultrastructural Research Team, RIKEN Center for Life Science Technologies, 2-2-3 Minatojima-minamimachi, Chuo-ku, Kobe, Hyogo 650-0047, Japan*

[5] *Department of Cell Biology, Tokushima University Graduate School of Medical Science, 3-18-15 Kuramoto-cho, Tokushima 770-8503, Japan*

[6] *Department of Chemistry, School of Science, The University of Tokyo, 7-3-1 Hongo, Bunkyo, Tokyo 113-0033, Japan*

[7] *Faculty of Life & Environmental Sciences, University of Tsukuba, 1-1-1 Tennodai, Tsukuba, Ibaraki, 305-8572 Japan*

[8] *Institut de Recherche XLIM, UMR CNRS No. 7252, 123 avenue Albert Thomas, 87060 Limoges CEDEX, France*

[9] *Institute of Applied Physics, University of Tsukuba, 1-1-1 Tennodai, Tsukuba, Ibaraki 305-8573, Japan*

[10] *Tsukuba Research Center for Interdisciplinary Materials Science (TIMS), University of Tsukuba, 1-1-1 Tennodai, Tsukuba, Ibaraki 305-8571, Japan*

†*Present address: National Research Institute of Police Science, 6-3-1, Kashiwanoha, Kashiwa, Chiba 277-0882, Japan*

Correspondence and requests for materials should be addressed to Akihito Inoko (ainoko@aichi-cc.jp) or Hideaki Kano(hkano@bk.tsukuba.ac.jp).

The PDF file includes

Supplementary Figure 1 | SHG, SFG, THG, TSFG, and TPEF processes.

Supplementary Figure 2 | Custom-made penta-modal coherent nonlinear optical microscope (CNOM).

Supplementary Figure 3 | Label-free visualization of rat retina by CNOM.

Supplementary Figure 4 | Polarization-dependence diversity of harmonophores.

Supplementary Figure 5 | SHG and TPEF from rat retina using 775 nm excitation.

Supplementary Figure 6 |Uncropped scans of immunoblots used in Fig. 5c.

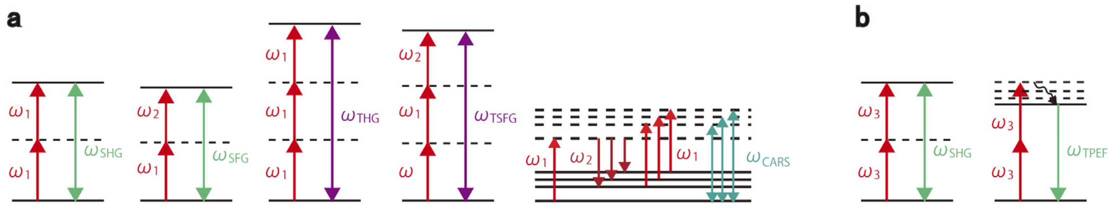

Supplementary Figure 1 |SHG, SFG, THG, TSFG, and TPEF processes. Five possible coherent nonlinear optical processes and one incoherent nonlinear optical process. **(a)** In the SHG process, two photons with angular frequency ω_1 combine to form one photon with twice the angular frequency ($2\omega_1$). Similar to SHG, in the SFG process, two photons with angular frequency ω_1 and ω_2 combine to form one photon with angular frequency $\omega_1+\omega_2$. The signals in the visible range indicate the sharp and broad spectral profiles, which correspond to SHG and SFG, respectively. As in SHG, in the THG process, three ω_1 photons combine to form a $3\omega_1$ (355 nm) photon. Also, like SFG, a $2\omega_1+\omega_2$ photon is created in the TSFG process. In the NIR region, the CARS process takes place, and a $2\omega_1$-ω_2 photon is created. These five nonlinear optical processes are coherent, whereas TPEF loses memory of coherence of the incident photons, i.e., it is an incoherent process **(b)**.

a (used in Fig. 1)

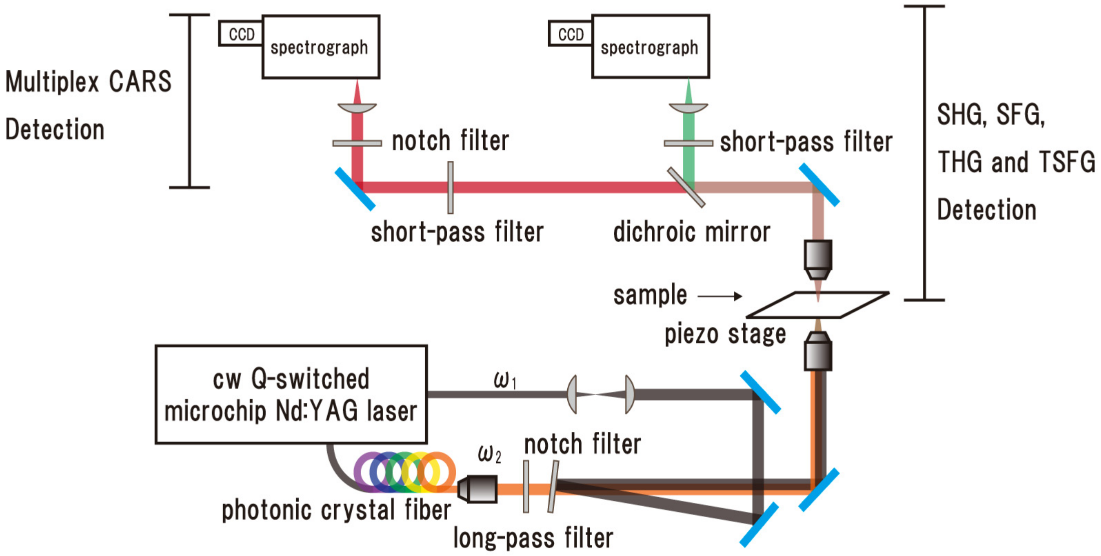

b (used in Fig. 2–5)

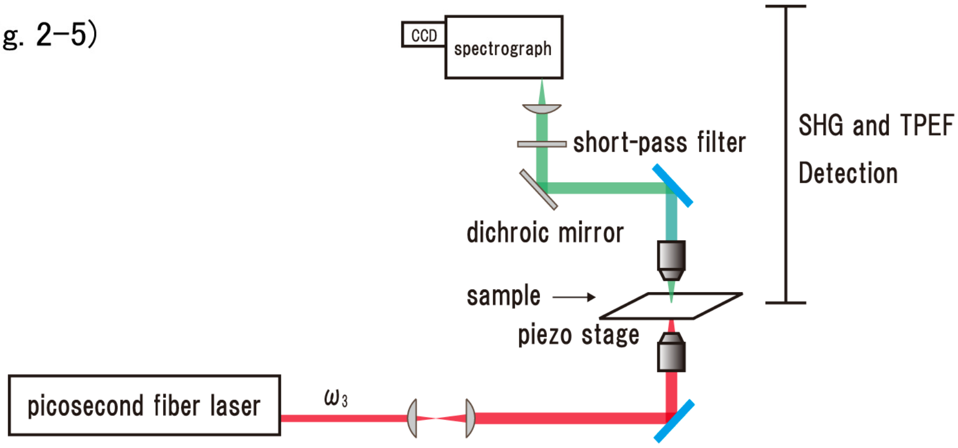

Supplementary Figure 2 |Custom-made penta-modal coherent nonlinear optical microscope (CNOM).

Penta-modal (SHG, CARS, SFG, THG, and TSFG) imaging system used in Fig. 1. The master laser source was a microchip Nd:YAG laser. The output from the master laser was divided in two. One portion was used directly as the pump pulse (or ω_1 pulse) for coherent and incoherent nonlinear optical processes such as CARS. The other was introduced into a photonic crystal fiber (PCF) to generate supercontinuum (SC), which was used as the Stokes pulse (or ω_2 pulse). Both the ω_1 and ω_2 pulses illuminated a sample mounted on the custom-made microscope. The sample was scanned to obtain images using a piezo stage. The signals from the sample were separately detected by two sets of spectrometers for UV-visible and NIR. **(b)** The bi-modal (SHG and TPEF) imaging system used in Fig. 2-5. We introduced another pulsed laser source with 775 nm excitation (ω_3 pulse) in the setup shown in **(a)**.

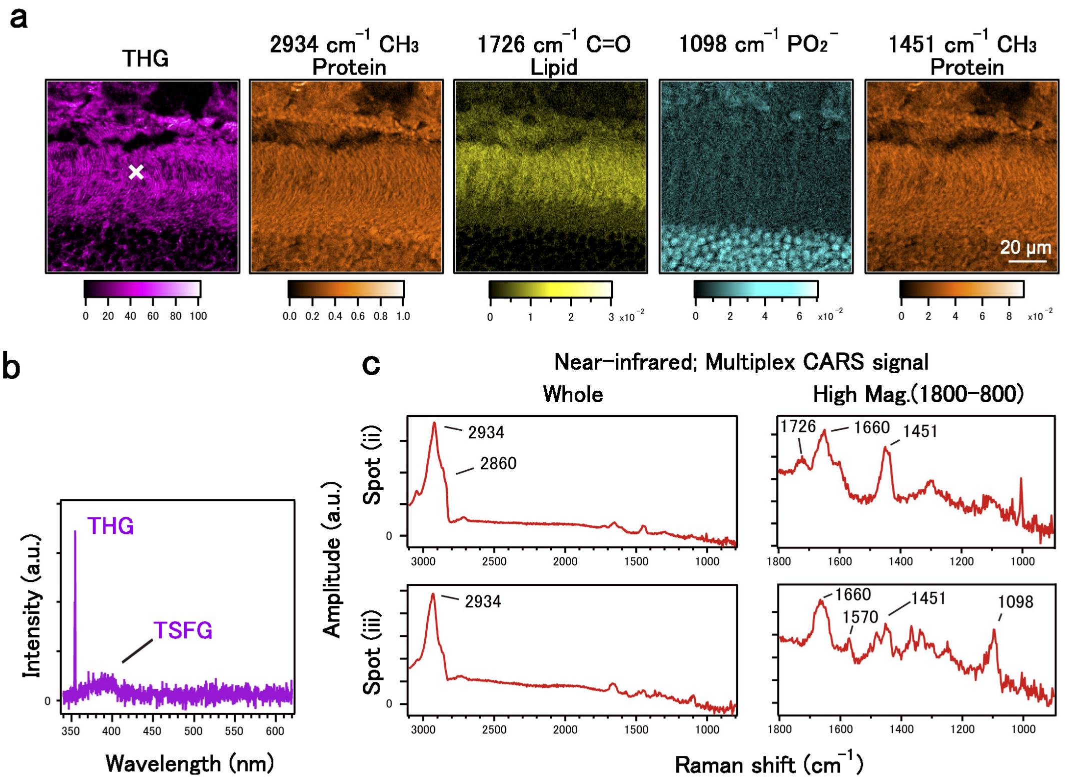

Supplementary Figure 3 | Label-free visualization of rat retina by CNOM. Other nonlinear optical spectroscopic images obtained by CNOM are summarized (a). The THG image gives high contrast at both the sclera and outer segment. The CARS images at 2934 cm^{-1} (CH_3 stretch vibrational mode) and 1451 cm^{-1} (CH_3 deform vibrational mode) visualize the distribution of protein, which is present throughout the tissue sample except around nuclei. The CARS image at 1726 cm^{-1} (C=O stretch vibrational mode due to the ester bond) visualizes the distribution of lipids, providing similar contrast to that at 2860 cm^{-1}. The CARS image at 1098 cm^{-1} (PO_2^- stretch vibrational mode due to DNA) gives similar contrast to that at 1570 cm^{-1}. (b) Representative spectra of THG and third-order sum frequency generation (TSFG). (c) In the representative spectra, protein-assignable CH_3 stretch, amide I, and CH3 deform vibrational modes were observed as bands at 2934, 1660, and 1451 cm^{-1}, respectively. In particular, position (ii) (indicating the photoreceptor layer) provided the bands at 2860 and 1726 cm^{-1}, assigned as CH_2 stretch and C=O stretch (ester) due to lipids. On the other hand, the position (iii) (indicating the outer nuclear layer) gave bands at 1570 and 1098 cm^{-1}, assigned as purine ring stretch due to adenine and guanine (1570 cm^{-1}) and PO_2^- due to nucleic acid (1098 cm^{-1}).

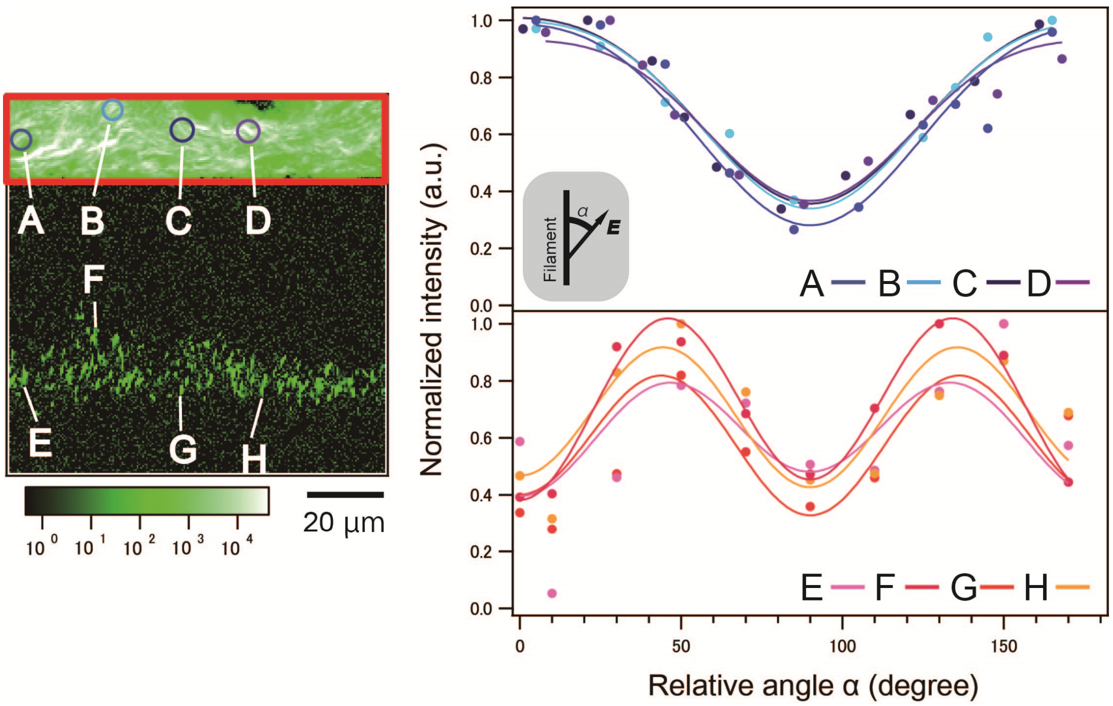

Supplementary Figure 4 |Polarization-dependence diversity of harmonophores. SHG image of retinal layers and its polarization dependence were analyzed in **(a)**. The SHG signal was observed at sclera due to collagen fibrils (top) and at photoreceptor cells due to Rootletin filaments (middle). By defining the angle (α) between incident laser polarization and the orientation of the filamentous structures, the polarization dependence of collagen and Rootletin were plotted in **(b)**. Note that the polarization dependence of the setup itself was corrected by dividing the signal intensity by the average of the SHG signal intensity, indicated by the red frame. Collagen gave an SHG signal maximum around 0 and 180 degrees. On the other hand, Rootletin gave a maximum around 45 and 135 degrees. These polarization dependences were fitted using the theoretical curve[46], and the harmonophore orientation angle with respect to the filament was calculated to be 55.5±3.6 degrees for Rootletin, close to that of myosin (62 degrees) [46] .

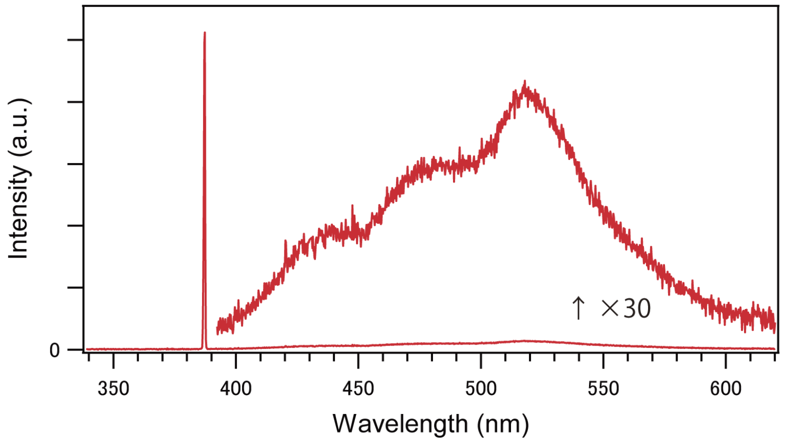

Supplementary Figure 5 | SHG and TPEF from rat retina using 775 nm excitation. Spectral profile of immunostained retina at the position of the SHG filament under 775 nm laser excitation. The sharp and intense band at 388 nm and the broad and weak band around 520 nm correspond to SHG and TPEF, respectively. The bands around 440, 520, and 475 nm were assigned as TPEF due to DAPI, Alexa Fluor 488, and auto-fluorescence, respectively. We first decomposed the band into three, and then the TPEF images of DAPI and Alexa Fluor 488 were reconstructed.

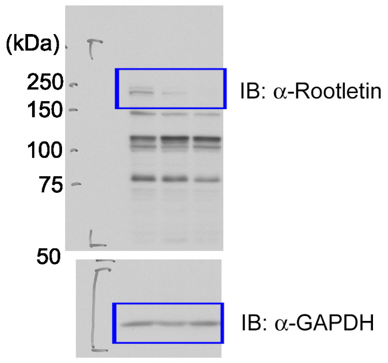

Supplementary Figure 6 | Uncropped scans of immunoblots used in Fig. 5c. Blue boxes indicate the cropped regions.
